# Supplementary material for: Negative feedback may suppress variation to improve collective foraging performance
Source: PLoS Comput Biol. 2022 May 18;18(5):e1010090. doi: 10.1371/journal.pcbi.1010090 (PMC9154117; doi:10.1371/journal.pcbi.1010090)
Supplement: S1 Text — (PDF) [file pcbi.1010090.s001.pdf]

Supplementary text of the article Negative feedback may suppress variation to improve collective foraging performance

Andreagiovanni Reina and James A. R. Marshall

S1 Text. Relationship between transition rates and food patch quality

Through numerical optimisation, we defined for both models the functional form of the transition rates that approximates best the target distribution. The optimisation routine computes the cumulative sum of errors of the mean-field model. The error is summed for all n food patches. This sum is also cumulative with respect to the time dimension, that is, the sum of errors is computed every time unit until the end of the simulation at $T_{\max}$, as:

$$\Theta = \sum_{t=1}^{T_{\max}} \sum_{i=1}^{n} \left| x_i^t - \frac{q_i}{\sum_{j \in n} q_j} \right| , \qquad \text{(SE1)}$$

where $|\,.\,|$ is the operator for absolute value. The optimisation routine explores the parameter space to find the values that minimise Θ of Eq. (SE1). In order to find parameters that would work well for different numbers of food patches n, different quality values, and for different initial conditions, we compute Θ (the cumulative sum of errors of Eq. (SE1)) for several setups, and use their sum as the value to minimise. We test both $n = 2$ and $n = 3$. For $n = 2$, we fix quality $q_2 = 0.5$ and compute Θ for $q_1 \in \{0, 0.1, 0.2, ..., 1.0\}$. For $n = 3$, we fix qualities $q_2 = 0.6$ and $q_2 = 0.3$, and compute Θ for $q_1 \in \{0, 0.1, 0.2, ..., 1.0\}$. We also test all possible initial states with sub-populations committed to the two, or three, options with step 0.1 (e.g., $(x_1, x_2, x_3) \in \{(0,0,0), (0,0,0.1), (0,0,0.2), ..., (0.2, 0.6, 0.1), ..., (1,0,0)\}$). The sum of Θ in all these setups is the quantity that is minimised by the optimisation routine. The optimisation code and results are available on GitHub at `https://github.com/DiODeProject/VarianceSuppression`.

We set the rates as logistic functions of the food patch quality,

$$\lambda = \frac{2\bar{\lambda}}{e^{-s(q_i - 0.5)} + 1} , \qquad \text{(SE2)}$$

where λ is a generic rate, $\bar{\lambda}$ is the average rate frequency, and s determines the slope of the curve[1]. Figure A shows some representative curves of Eq. (SE2) for different values of the slope s. The optimisation routine selects values in the range $s \in [0, 5]$, representing at the two extremes the constant function ($s = 0$, quality-insensitive), and approximately a linear relationship ($s = 5$, $\lambda \approx 2\bar{\lambda}q_i$).

We run the optimisation routine in a variety of configurations to determine both the best relative strength between the transition rates (rate strength $\bar{\lambda} \in (0, 1000]$ for abandonment, recruitment, and stop signalling) and their functional form (slope s for all rates). The process speed is always normalised on the average rate of discovery, which is fixed at value $\bar{\lambda} = 1$ in every test. The results are reported in Table A to F for various configurations. These results motivated the definition of the two streamlined models that we presented in the main text, as indicated in the following.

Strong recruitment is most beneficial when combined with stop signalling. In Table A, we allowed the optimisation routine to freely select the best average rate strengths and slope for all rates. Instead in Table B, we run the same process but fixed stop-signalling strength to zero (positive feedback only). When stop signalling was possible, the optimised recruitment reached values

[1] Note that the abandonment rate can be inversely proportional to the food patch quality, therefore the term q_i is replaced by $(1 - q_i)$.

$T_{\max}$	Discovery		Recruitment		Abandonment		Stop signalling		error Θ
	$\bar{\lambda}$	slope s	$\bar{\lambda}$	slope s	$\bar{\lambda}$	slope s	$\bar{\lambda}$	slope s	
10	1	3.18753	993.266	3.55255	1.50558	1.64438	25.8788	0.00355132	0.167723
100	1	4.01337	990.897	3.59278	0.722135	1.57534	12.811	0.	1.61001
1000	1	4.86449	973.061	3.66919	0.229161	1.86251	4.84565	0.	15.5916

Table A: Optimised parameters with all rates and slopes free (expect for discovery $\bar{\lambda} = 1$).

$T_{\max}$	Discovery		Recruitment		Abandonment		Stop signalling		error Θ
	$\bar{\lambda}$	slope s	$\bar{\lambda}$	slope s	$\bar{\lambda}$	slope s	$\bar{\lambda}$	slope s	
10	1	4.0017	3.82747	1.97406	0.862282	0.00372217	0	0	0.71383
100	1	4.34572	0.776195	1.94331	0.166382	0.138089	0	0	3.60998
1000	1	4.09265	11.2862	0.0360765	0.294101	0.0811387	0	0	21.5086

Table B: Optimised parameters with all rates and slopes free, except for discovery strength fixed to 1 and stop-signalling strength fixed to zero, i.e. model without negative feedback.

$T_{\max}$	Discovery		Recruitment		Abandonment		Stop signalling		error Θ
	$\bar{\lambda}$	slope s	$\bar{\lambda}$	slope s	$\bar{\lambda}$	slope s	$\bar{\lambda}$	slope s	
10	1	4.50084	100.	3.75795	0.481693	2.57493	8.09239	0.0495871	0.240978
100	1	4.85832	100.	3.70402	0.206428	2.04744	3.8593	0.0131419	1.85758
1000	1	4.61453	100.	3.56596	0.0947592	1.82524	1.3963	0.0672265	16.3936

Table C: Optimised parameters with recruitment strength fixed to 100 and discovery strength $\bar{\lambda} = 1$. Values of all other rates and slopes are free.

$T_{\max}$	Discovery		Recruitment		Abandonment		Stop signalling		error Θ
	$\bar{\lambda}$	slope s	$\bar{\lambda}$	slope s	$\bar{\lambda}$	slope s	$\bar{\lambda}$	slope s	
10	1	3.65107	100.	0.0361097	9.99617	0.0463419	0	0	0.845349
100	1	4.04299	100.	0.0268109	5.74431	0.00317431	0	0	3.93436
1000	1	4.02302	100.	0.0108346	2.12533	0.00579289	0	0	21.825

Table D: Optimised parameters with recruitment strength fixed to 100, stop-signalling strength fixed to zero, and discovery strength fixed to 1. Values of all other rates and slopes are free.

$T_{\max}$	Discovery		Recruitment		Abandonment		Stop signalling		error Θ
	$\bar{\lambda}$	slope s	$\bar{\lambda}$	slope s	$\bar{\lambda}$	slope s	$\bar{\lambda}$	slope s	
10	1	5.	1.	5.	0.171573	5.	0.586042	0.	0.619
100	1	4.97243	1.	3.74725	0.076198	1.96719	0.176224	0.110512	3.19643
1000	1	4.42046	1.	3.11191	0.0413467	0.879217	0.0486974	0.00521406	20.1137

Table E: Optimised parameters with both recruitment and discovery strengths fixed to $\bar{\lambda} = 1$. Values of all other rates and slopes are free.

$T_{\max}$	Discovery		Recruitment		Abandonment		Stop signalling		error Θ
	$\bar{\lambda}$	slope s	$\bar{\lambda}$	slope s	$\bar{\lambda}$	slope s	$\bar{\lambda}$	slope s	
10	1	4.53101	1.	4.92961	0.500082	0.321492	0	0	0.719155
100	1	4.35149	1.	1.82299	0.180247	0.0761111	0	0	3.61027
1000	1	4.09204	1.	1.04416	0.0643803	0.117602	0	0	20.9055

Table F: Optimised parameters with both recruitment and discovery strengths fixed to $\bar{\lambda} = 1$, and stop-signalling fixed to zero. Values of all other rates and slopes are free.

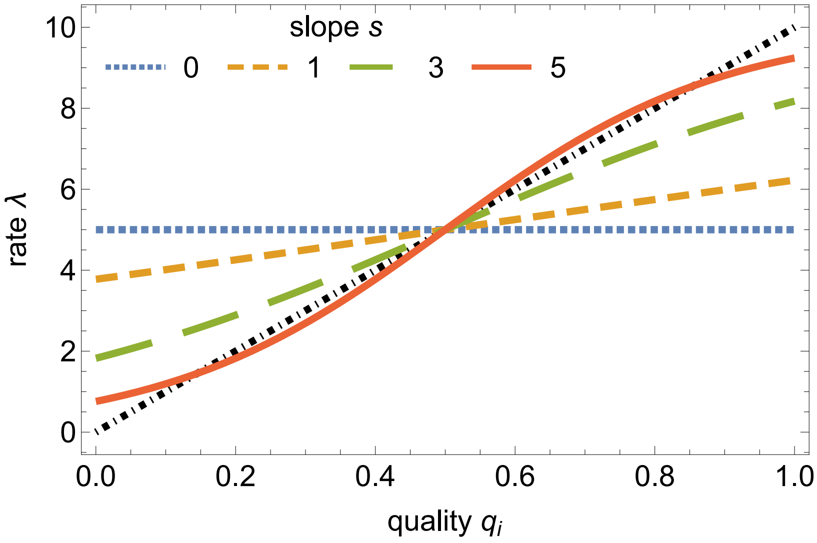

Figure A: The relationship between the food patch quality q_i (x-axis) and the transition rate λ (y-axis) is modelled with Eq. (SE2). We report four representative examples for $\bar{\lambda} = 5$ and slope values $s \in \{0, 1, 3, 5\}$. With $s = 0$ (blue dotted line), the rate is quality-independent, *i.e.* $\lambda = \bar{\lambda}$; instead, with $s = 5$ (orange solid curve), the relationship between λ and q_i is approximately linear (the linear function $\lambda = 2\bar{\lambda}q_i$ is included for comparison as a black dot-dashed line).

close to the highest boundary ($\sim$ 1000). Instead, the system with disabled negative feedback (*i.e.* stop signalling $\bar{\lambda} = 0$) set recruitment to values two to three orders of magnitude smaller than when inhibition is enabled. Comparing the errors resulting from the two configurations, for every tested $T_{\max}$ (*i.e.* quick or slow dynamics), the system with both positive and negative feedback always has a lower error Θ.

Recruitment is proportional to quality only when there is negative feedback. In Table C and D, we fixed the recruitment strength to a high value, 100, and optimised all other parameters for the system with and without negative feedback, respectively. We can see that in the system without negative feedback (Table D), the optimised slope for recruitment has values close to 0, therefore recruitment is quality-insensitive. On the contrary, the system with negative feedback (Table C) has high values of slope s, approximating a linear relationship. Therefore, in the two models of the main text, we selected the relationships that minimised the error and could best approximate the target distribution: we set the recruitment rate in the model with positive feedback only as constant, and in the model with negative feedback as linearly proportional to the quality q_i.

Stop signalling is relatively small and constant. In Tables A, C, and E, we can see that stop signalling strength $\bar{\lambda}$ is always one to two orders of magnitude smaller than recruitment and, most importantly, always has a slope close to zero. A slope close to zero means that the best results are given when stop signalling is constant and independent of the quality value. Therefore, in the main text, the model has constant stop signalling, with strength optimised to the recruitment strength.
