# Supplementary material for: Negative feedback may suppress variation to improve collective foraging performance
Source: PLoS Comput Biol. 2022 May 18;18(5):e1010090. doi: 10.1371/journal.pcbi.1010090 (PMC9154117; doi:10.1371/journal.pcbi.1010090)
Supplement: S2 Text — (PDF) [file pcbi.1010090.s002.pdf]

Supplementary text of the article
Negative feedback may suppress variation to improve collective foraging performance

Andreagiovanni Reina and James A. R. Marshall

S2 Text. Parameters of the models

In our study, the quality q_i is normalised in the range $q_i \in [0, 1]$. The abandonment rate is set to a constant low value $a = 10^{-3}$. The two models have different recruitment rates r_i: the model with negative feedback scales linearly with the option's quality, *i.e.* $r_i = \rho q_i$, and the model without negative feedback has a quality-independent rate $r_i = \rho$. The scaling factor ρ that tunes the recruitment strength is set in order to have the same average rate r in both models. Therefore, assuming that the expected quality $E(q)$ is the average of the range $[0, 1]$, thus $E(q) = 0.5$, we have that ρ in the model without negative feedback is half of ρ in the model with negative feedback. In every plot of this paper, we indicate r, the average value of the possible rates r_i. The self-inhibition strength z is tuned through numerical simulation to the best value in terms of speed and accuracy. In particular, we computed the strength of z which took the system to a sum of squared error $SSE < 10^{-4}$. The squared error is computed as the sum of the squared distance from the target distribution for every population. Figure A shows that the best stop signalling strength increases with the positive social feedback ρ. Figure 3 of the main text shows how the stop signalling strength influences the speed and the robustness of the system. As documented in previous work [1, 2], stronger signalling leads to quicker but less robust, or less accurate, dynamics.

References

[1] Pais D, Hogan PM, Schlegel T, Franks NR, Leonard NE, Marshall JAR. A Mechanism for Value-Sensitive Decision-Making. PLoS ONE. 2013;8(9):e73216.

[2] Reina A, Marshall JAR, Trianni V, Bose T. Model of the best-of-N nest-site selection process in honeybees. Physical Review E. 2017;95(5):052411.

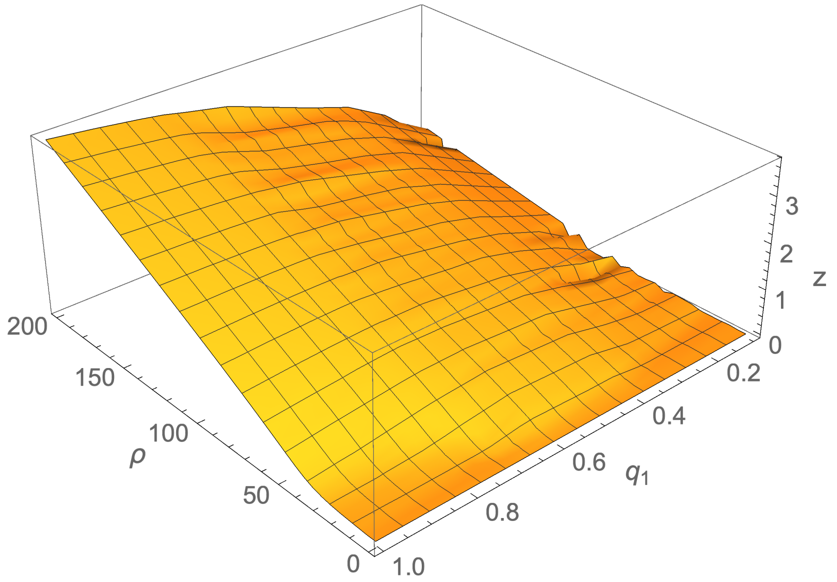

Figure A: The negative social feedback is numerically computed for each tested value of positive social feedback strength ρ and food patch quality q_1 (for two-patches environments and $q_2 = 0.5$). The optimal negative social feedback is linearly proportional to ρ and q_1.
