# Supplementary material for: Negative feedback may suppress variation to improve collective foraging performance
Source: PLoS Comput Biol. 2022 May 18;18(5):e1010090. doi: 10.1371/journal.pcbi.1010090 (PMC9154117; doi:10.1371/journal.pcbi.1010090)
Supplement: S3 Text — (PDF) [file pcbi.1010090.s003.pdf]

Supplementary text of the article
Negative feedback may suppress variation to improve collective foraging performance

Andreagiovanni Reina and James A. R. Marshall

S3 Text. Stability analysis

The dynamics of the two systems, described in the main text through chemical reactions (see Table 1 in the main text), can also be described via ODE systems. The system without negative social feedback reads as:

$$\begin{aligned} \dot{x_i} &= q_i\, x_U + x_i(\rho\, x_U - a), \qquad i \in \{1, \ldots, n\} \\ x_U &= 1 - \textstyle\sum_{i=1}^{n} x_i \,, \end{aligned} \tag{SE1}$$

where x_i is repeated for the n subpopulations committed to the n patches. The system of Eq. (SE1) has two fixed points. Only one of the points is stable and exists in the solution domain relevant to our study, *i.e.* within $0 \leq x_i^* \leq 1$. This point is

$$x_i^* = \frac{q_i}{\sum_{j=1}^{n} q_j}\, \epsilon \,, \tag{SE2}$$

where $\epsilon \approx 1$, for small leak $a \ll 1$. Therefore, the expected solution accurately approximates the target distribution of Eq. (1) in the main text.

The n eigenvalues of the system of Eq. (SE1) at the stable fixed point of Eq. (SE2) are all negative. However, one of the eigenvalues has the absolute value several order of magnitude larger than the value of the other $n-1$ eigenvalues which are approximately equal to zero. This asymmetry in the eigenvalue strength explains the type of dynamics observed in the system (*e.g.* Figure 1 bottom-left of the main text). The population quickly gets committed to the n food patches, *i.e.* with $x_u \approx 0$, and then imperceptibly slowly moves towards the point of Eq. (SE2) on the $(n-1)$-dimension plane $x_1 = x_2 = \cdots = x_n$. However, the attraction is so low that practically the system does not move once it reaches that plane.

The mean-field model of the system with negative social feedbacks is described by the following ODE system:

$$\begin{aligned} \dot{x_i} &= q_i\, x_U + x_i(\rho\, q_i\, x_U - a - z\, x_i), \qquad i \in \{1, \ldots, n\} \\ x_U &= 1 - \textstyle\sum_{i=1}^{n} x_i \,. \end{aligned} \tag{SE3}$$

We could not find the analytical solution for the system of Eq. (SE3) however numerical integration (*e.g.* see Figure 1 top-right of the main text) shows that the system can asymptotically converge to values very close to the target distribution of Eq. (1) of the main text. In all our simulations, through numerical optimisation we identified the best value of z in terms of rapid convergence and small error (see more details in the Section *Parameters* below). Similarly to the system of Eq. (SE1), also the system of Eq. (SE3) has eigenvalues with different magnitude, however here the attraction is strong enough to move the system towards the fixed point in a finite time.

Comparing the eigenvalues of the two systems, the magnitude of the dominant negative eigenvalue (*i.e.* with largest magnitude) is of comparable size among the two systems. For the system without negative social feedback, the value is approximately $-r$, while for the system with negative feedback is approximately $-\rho\, \bar{q}$ (with $\bar{q}$ being the average food patch quality). Instead, the second dominant negative eigenvalues differ of several orders of magnitude between the two systems. For example, for the parameters used in Figure 1 of the main text, the eigenvalue of the system without negative feedback is about -10^{-5}, while in the system with negative feedback is about -1.4.

Through the MuMoT software [1], we derived the Fokker-Planck equation of both systems and the second-order moments of the noise for the stochastic system with finite size (see the Jupyter notebook

available with to the paper[1]). The noise equations are difficult to solve analytically therefore we applied simplifying assumptions to obtain a first order approximation. We set to zero—because small and hence we assume have small impact—the proportion of uncommitted individuals at convergence x_u^* and the abandonment rate a. We obtain that, at convergence, $t \to \infty$, the noise's variance for one the two foraging subpopulations, x_1, in the system without negative social feedback is $\langle \eta_1^2 \rangle = \frac{x_1^*}{2}$. Because the system with negative social feedback is mathematically more complex, we also need to assume that at convergence the covariance $\langle \eta_1 \eta_U \rangle$ is zero. With these assumptions, the noise's variance for one the two foraging subpopulations in the system with negative social feedback is $\langle \eta_1^2 \rangle = \frac{x_1^*}{4}$. Therefore, these results confirm the results we obtained computationally, that is, the noise variance is higher in the system without negative social feedback.

References

[1] Marshall JAR, Reina A, Bose T. Multiscale Modelling Tool: Mathematical Modelling of Collective Behaviour without the Maths. PLoS ONE. 2019;14(9):e0222906.

[2] Jupyter P, Bussonnier M, Forde J, Freeman J, Granger B, Head T, et al. Binder 2.0 - Reproducible, interactive, sharable environments for science at scale. In: Proceedings of the 17th Python in Science Conference; 2018. p. 113-20.

[1] The notebook is available at `https://github.com/DiODeProject/MuMoT/blob/master/DemoNotebooks/Variance\_suppression.ipynb` and also accessible in the interactive executable version on Binder [2] at https://mybinder.org/v2/gh/DiODeProject/MuMoT/master?filepath=DemoNotebooks%2FVariance_suppression.ipynb.
