# Supplementary material for: Negative feedback may suppress variation to improve collective foraging performance
Source: PLoS Comput Biol. 2022 May 18;18(5):e1010090. doi: 10.1371/journal.pcbi.1010090 (PMC9154117; doi:10.1371/journal.pcbi.1010090)
Supplement: S4 Text — (PDF) [file pcbi.1010090.s004.pdf]

Supplementary text of the article
Negative feedback may suppress variation to improve collective foraging performance

Andreagiovanni Reina and James A. R. Marshall

S4 Text. Variance reduction in a large parameter space

We tested the two systems with a wide range of parameters in order to show that the results presented in the main text for specific values hold in a larger parameter space. Figure A shows the variance of each model for varying the average recruitment strength r (positive social feedback) and food patch quality. The reported variance is computed as

$$\text{Variance}(x_1 + x_2) = \text{Variance}(x_1) + \text{Variance}(x_2) + 2\,\text{Covariance}(x_1, x_2) \qquad \text{(SE1)}$$

for the case of $n = 2$ food patches.

Figure B shows the ratio between the variance of the system without negative feedback over the variance of the system with negative feedback. The ratio never goes below one and thus shows that using social negative feddback always reduces variance, in the tested systems.

Similarly, Figure C shows how the variances are influenced by the swarm size S.

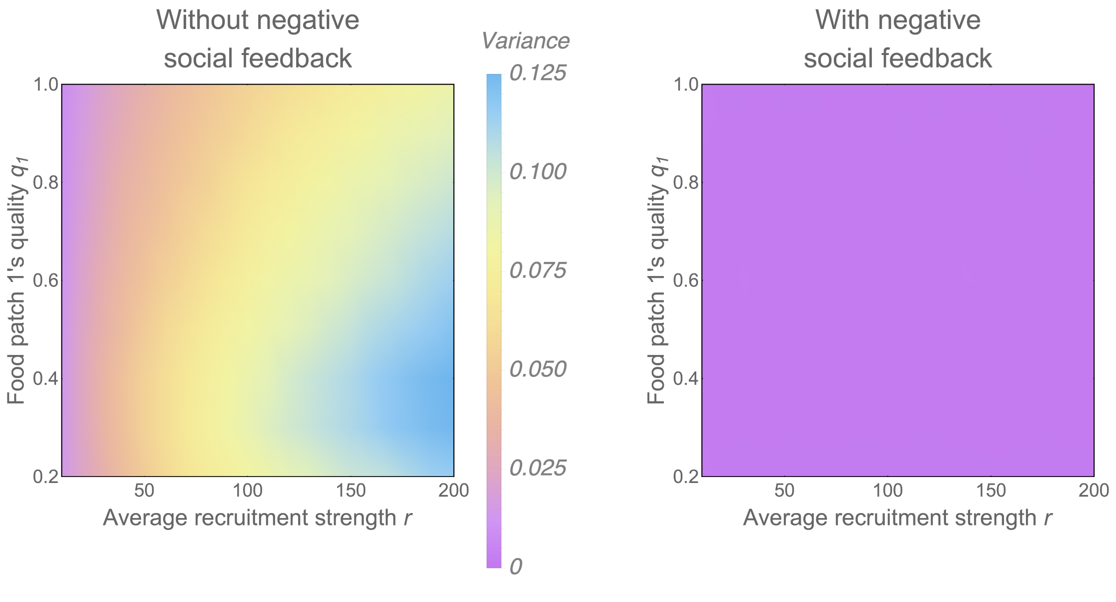

Figure A: Variance of the model without (left) and with (right) negative social feedback for varying average recruitment strength r (x-axis) and food patch quality (y-axis). The variance is computed using Eq. (SE1). The system is simulated through SSA and we report the average of 1000 runs. The tests are for swarm size $S = 200$ and $n = 2$ food patches, with food patch 2's quality $q_2 = 0.5$ and food patch 1's quality varied on the y-axis in $[0.2, 1]$. In the full space the variance of the model with negative social feedback (right panel) is considerably lower than the variance of the other model's variance.

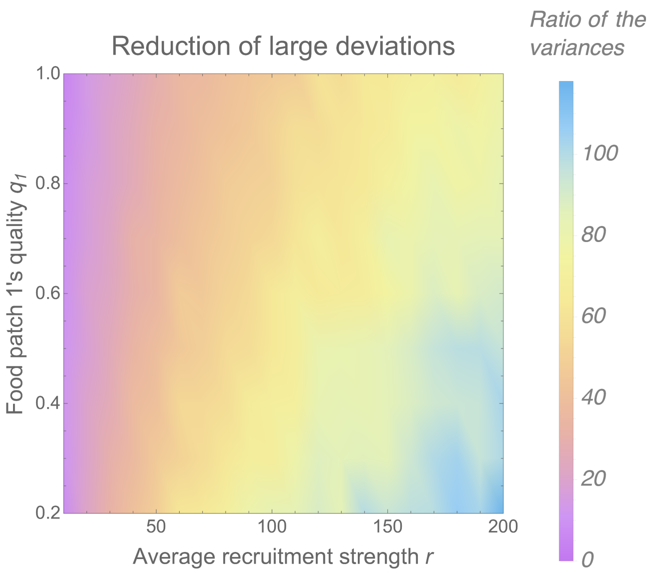

Figure B: Ratio of the variance of the system without and with negative social feedback (from Fig. A). The variance is computed using Eq. (SE1). The ratio never goes under one showing that the system with negative social feedback has always variance lower than the variance of the system without negative social feedback.

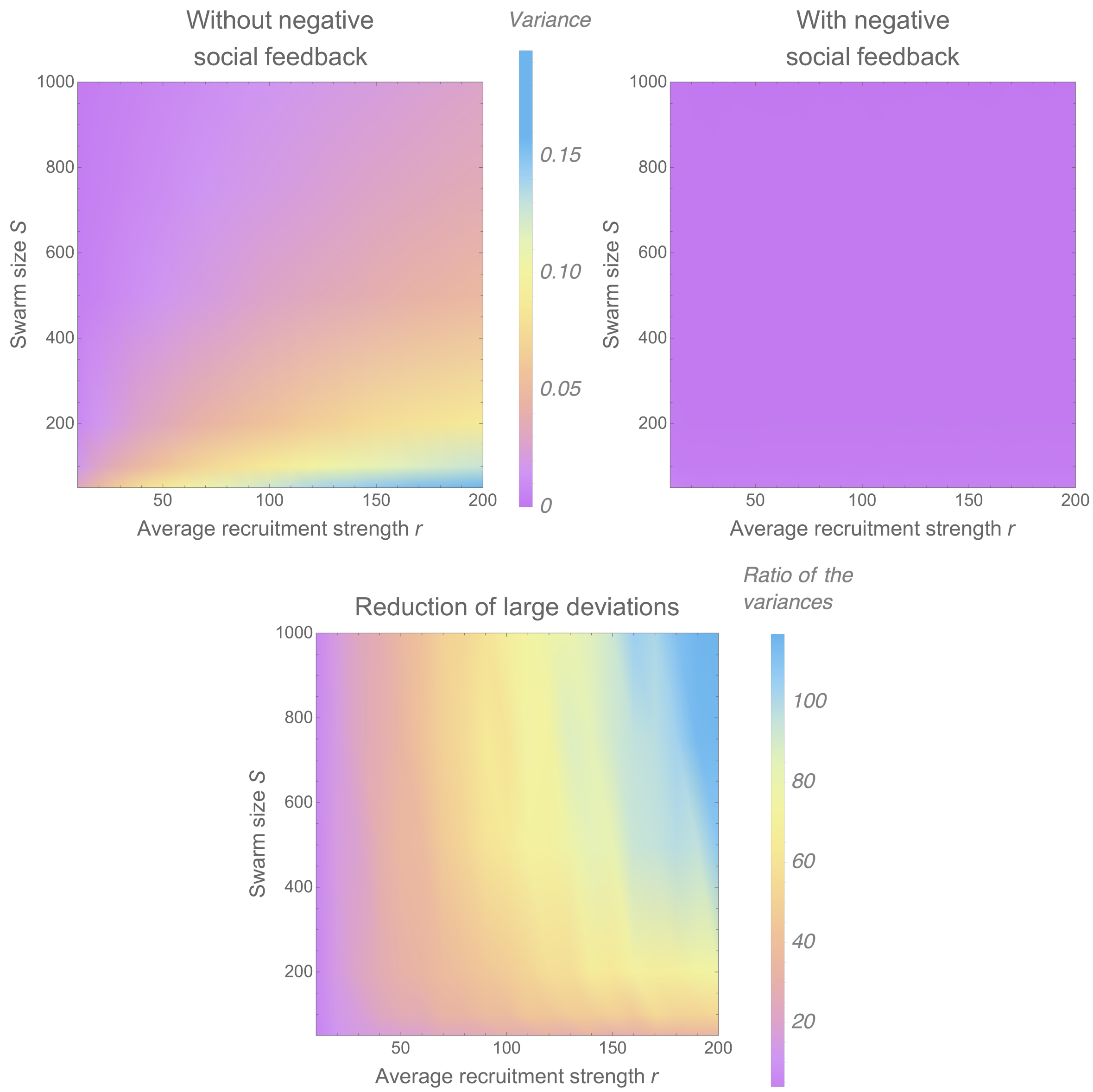

Figure C: Variance of the system for varying swarm size $S \in [50, 1000]$ and average recruitment strength $r \in [10, 200]$ for $q_1 = 1$ and $q_2 = 0.5$.. For any tested swarm size S, we observe the same pattern described in the main text.
