# Supplementary material for: Negative feedback may suppress variation to improve collective foraging performance
Source: PLoS Comput Biol. 2022 May 18;18(5):e1010090. doi: 10.1371/journal.pcbi.1010090 (PMC9154117; doi:10.1371/journal.pcbi.1010090)
Supplement: S5 Text — (PDF) [file pcbi.1010090.s005.pdf]

Supplementary text of the article
Negative feedback may suppress variation to improve collective foraging performance

Andreagiovanni Reina and James A. R. Marshall

S5 Text. Effect of asocial negative feedback (abandonment or leak α)

We tested the effect of increasing the negative feedback at the individual level (*i.e.* abandonment independent of social interaction) on the collective response. In the analyses reported in the main text and in the other supplementary texts we used a constant abandonment rate $\alpha = 10^{-3}$. Figure A shows the stationary distribution of the subpopulations committed to the $n = 3$ food patches (same setup of Figure 1 of the main text) for different values of the abandonment rate $\alpha \in \{10^{-3}, 10^{-2}, 10^{-1}, 1, 10\}$ in the model without negative social feedback. While the ODE analysis predicts minimal deviations from the target distribution (see results of the stability analysis in S3 Text, and its Eq. (SE2)), the stochastic simulations evince high levels of variability for every tested value of α. Therefore, our analysis suggests that, despite being a form of negative feedback, asocial leaking (abandonment) is not sufficient to guarantee low levels of variance in the collective distribution.

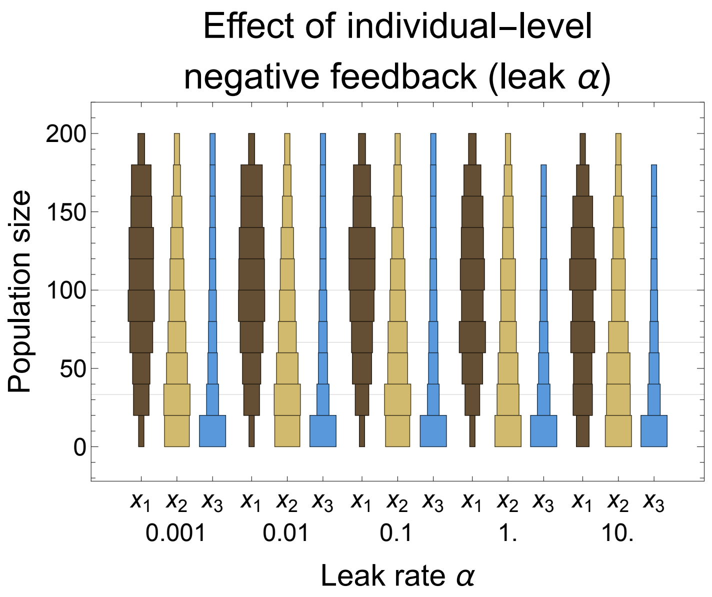

Figure A: Results from 10^3 SSA simulations of the model without negative social feedback for different values of the abandonment rate α (leak), changed on the x-axis. We ran simulations of swarms composed of $S = 200$ individuals operating in an environment with $n = 3$ food patches, with the same quality values used for Figure 1 of the main text, ($q_1 = 0.75, q_2 = 0.5, q_3 = 0.25$), and the average recruitment strength is $r = 100$. The distribution charts display how the population is divided, at the final step (at time 10), among the three sub-populations foraging from the different patches (x_1, x_2, x_3), labelled on the axis and represented with three different colours. Variance remains high also for high levels of independent (asocial) negative feedback.
