# Supplementary material for: Negative feedback may suppress variation to improve collective foraging performance
Source: PLoS Comput Biol. 2022 May 18;18(5):e1010090. doi: 10.1371/journal.pcbi.1010090 (PMC9154117; doi:10.1371/journal.pcbi.1010090)
Supplement: S6 Text — (PDF) [file pcbi.1010090.s006.pdf]

Supplementary text of the article
Negative feedback may suppress variation to improve collective foraging performance

Andreagiovanni Reina and James A. R. Marshall

S6 Text. Adapting to changing conditions

When the starting point is different from the symmetric point $\{x_1, x_2, x_3\} = \{0, 0, 0\}$, the system without negative social feedback has very slow dynamics compared to the system with social negative feedback. An example is shown in Figure A which shows the temporal evolution of the mean-field model for the starting point $\{x_1, x_2, x_3\} = \{0, 0.5, 0.5\}$. Such situations can happen when a change in the environment occur. While the system with negative social feedback reaches convergence with constant speed, unaffected by the starting point, the system without negative social feedback can only adapt in very long time.

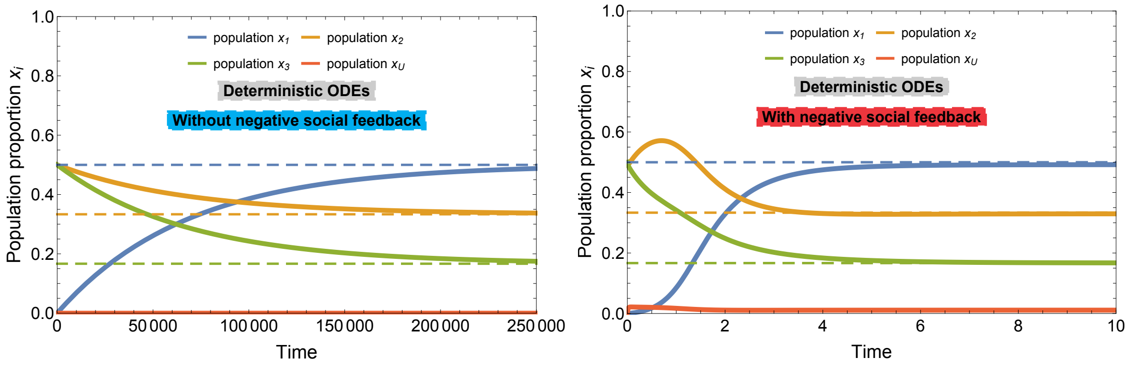

Figure A: ODE dynamics over time for starting point $\{x_1, x_2, x_3\} = \{0, 0.5, 0.5\}$. The system with negative social feedback (right) converges to the target distribution in a similar amount of time for every starting point. Instead, the system without negative social feedback (left) has very slow dynamics and the time to converge to the target distribution is more than five orders of magnitude larger than the symmetric case presented in the main text (Figure 1).
