# Supplementary material for: Negative feedback may suppress variation to improve collective foraging performance
Source: PLoS Comput Biol. 2022 May 18;18(5):e1010090. doi: 10.1371/journal.pcbi.1010090 (PMC9154117; doi:10.1371/journal.pcbi.1010090)
Supplement: S7 Text — (PDF) [file pcbi.1010090.s007.pdf]

Supplementary text of the article
Negative feedback may suppress variation to improve collective foraging performance

Andreagiovanni Reina and James A. R. Marshall

S7 Text. Sum of squared error

We measure the performance of the system at convergence by computing the sum of squared error (SSE). The SSE is computed as

$$SSE = \sum_{i=1}^{n} \left(x_i^T - \frac{q_i}{\sum_{j \in n} q_j} \right)^2 . \tag{SE1}$$

where x_i^T is the subpopulation committed to patch i at convergence time $T = 1000$.

Figure 2 of the main text shows how SSE—that is, the sum of the errors for each food source in achieving the target distribution—is significantly higher without negative social feedback.
