# Supplementary material for: Negative feedback may suppress variation to improve collective foraging performance
Source: PLoS Comput Biol. 2022 May 18;18(5):e1010090. doi: 10.1371/journal.pcbi.1010090 (PMC9154117; doi:10.1371/journal.pcbi.1010090)
Supplement: S8 Text — (PDF) [file pcbi.1010090.s008.pdf]

Supplementary text of the article
Negative feedback may suppress variation to improve collective foraging performance

Andreagiovanni Reina and James A. R. Marshall

S8 Text. Asocial model

By setting $\rho = 0$ in the model of Eq. (SE1) of S3 Text, we remove any social component and the system only relies on individual discovery of the food patches and spontaneous abandonment. Figure A shows that such a system has low variance centred around the target distribution. Additionally, the temporal dynamics, for the symmetric initial condition $\{x_1, x_2, x_3\} = \{0, 0, 0\}$, are comparable to the other two systems investigated in this study. The dynamics are slower when the starting point is different from $\{x_1, x_2, x_3\} = \{0, 0, 0\}$. Figure B illustrates an example for the starting point $\{x_1, x_2, x_3\} = \{0, 0.5, 0.5\}$. We note, however, that the system can speed up its dynamics by increasing the leak rate (*i.e.* spontaneous abandonment a) as illustrated in the right panel of Figure B. The spontaneous abandonment can be increased however not unboundedly as the parameter a impacts on how close to the target distribution the system converges to. In fact, the predicted fixed point for $\rho = 0$ in the model of Eq. (SE1) of S3 Text is:

$$x_i^* = \frac{q_1}{a + q_1 + q_2} \,. \qquad \text{(SE1)}$$

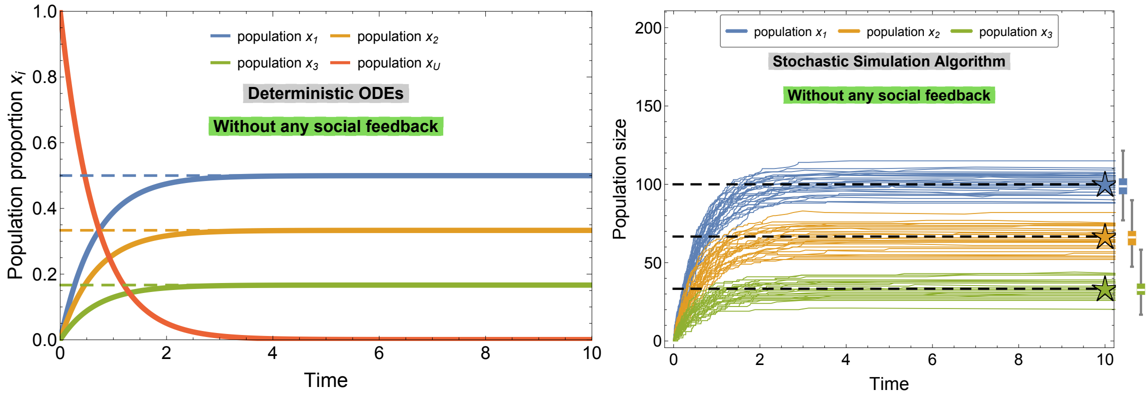

Figure A: Dynamics of the ODE model (left) and SSA simulations (right) of the system without social feedback with starting point $\{x_1, x_2, x_3\} = \{0, 0, 0\}$. The system without social feedback also displays small variance (boxplots on the right for 1000 simulations), however the dynamics can be slow for different starting points, *e.g.* see Figure B.

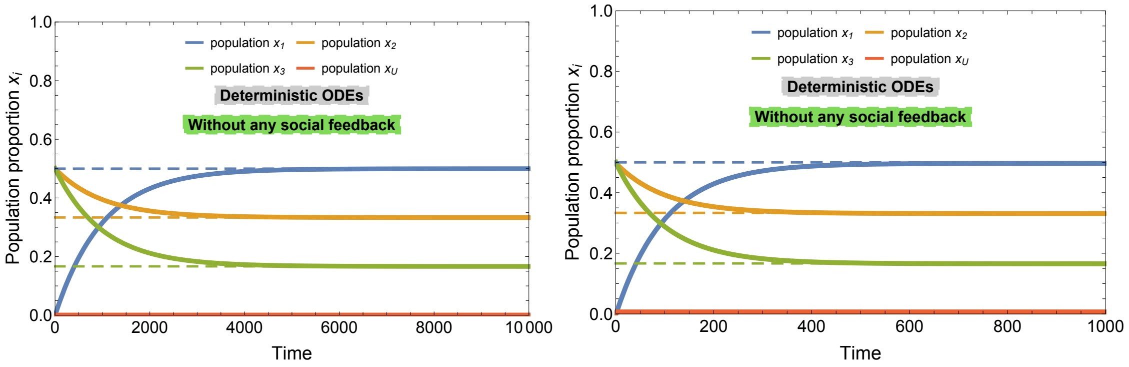

Figure B: ODE dynamics of the system without social feedback with starting point $\{x_1, x_2, x_3\} = \{0, 0.5, 0.5\}$. In both cases, the convergence time is longer than the symmetric condition of Figure A. On the left panel, the leak rate $a = 10^{-3}$ is the same of the other analysed models. On the right panel, we show that the system can speed up the dynamics by increasing the leak rate to $a = 10^{-2}$.
