# Supplementary material for: Negative feedback may suppress variation to improve collective foraging performance
Source: PLoS Comput Biol. 2022 May 18;18(5):e1010090. doi: 10.1371/journal.pcbi.1010090 (PMC9154117; doi:10.1371/journal.pcbi.1010090)
Supplement: S9 Text — (PDF) [file pcbi.1010090.s009.pdf]

Supplementary text of the article
Negative feedback may suppress variation to improve collective foraging performance

Andreagiovanni Reina and James A. R. Marshall

S9 Text. Large deviation from target

Figure B shows the probability that the state of the system at convergence would deviate from the target. We set an acceptance margin m and we measure the probability of laying outside such a margin. The results show the proportion of 1000 SSA runs where at least one of the subpopulations committed to either of the $n = 2$ food patches has a deviation larger than $m\,S$ (where m is the given margin and S the system size). In Figure B, we vary the margin value m and show that the probability of being outside any given margin is higher without negative social feedback.

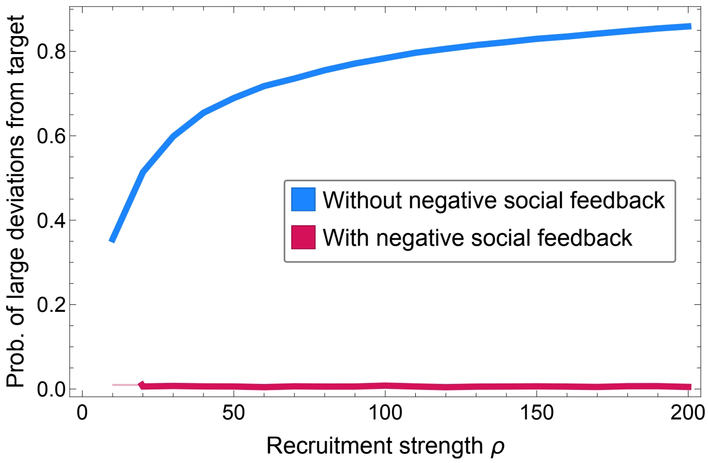

Figure A: Probability of large deviations from the target distribution for a system of $S = 200$ individuals computed from 10^3 SSA simulations. The probability is computed as the percentage of simulations that have the final population distribution with a deviation larger than $10\%S$ from the target distribution. The results are an average for $q_1 \in [0.2, 1]$ and $q_2 = 0.5$. The system with negative feedback shows a large reduction of the probability of large deviations. (95% confidence intervals are smaller of the line width.)

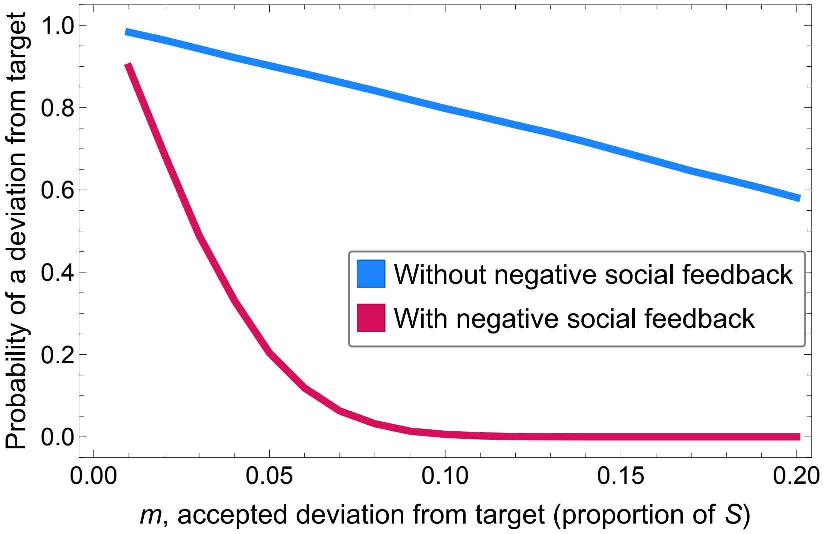

Figure B: We compute the probability of a deviation from the target distribution larger than a threshold m, where m is varied on the x-axis and the swarm size is $S = 200$. We report here the proportion of 10^3 SSA runs that had a deviation larger than m (y-axis) for an average recruitment strength $r = 100$. The system with negative social feedback has always a lower probability than the system without negative social feedback. (95% confidence intervals are smaller of the line width.)
