# Supplementary material for: Negative feedback may suppress variation to improve collective foraging performance
Source: PLoS Comput Biol. 2022 May 18;18(5):e1010090. doi: 10.1371/journal.pcbi.1010090 (PMC9154117; doi:10.1371/journal.pcbi.1010090)
Supplement: Attachment — Submitted filename: Reply_to_reviewers.pdf [file pcbi.1010090.s010.pdf]

UNIVERSITÉ LIBRE DE BRUXELLES

IRIDIA

Institut de Recherches Interdisciplinaires
et de Développements en Intelligence Artificielle

Dr Andreagiovanni Reina
IRIDIA, Université Libre de Bruxelles
Avenue F. Roosevelt 50
1050 Brussels
Belgium

Email: andreagiovanni.reina@ulb.be

PLOS Computational Biology Editorial Office

Subject: Revision of the submission PCOMPBIOL-D-22-00055

22nd March 2022

Dear Editor and Reviewers,

Thank you for for the time dedicated to read and evaluate our article. We appreciated the overall positive evaluation of the study and we are thankful for all the helpful comments on the previous version of the manuscript. We followed your suggestions in preparing the revised version. We reply in detail to each point.

Reviewer #1

> *I did find table 1 a bit confusing, because at first reading, it seemed to suggest that the negative-feedback model also has an additional change to the positive feedback process, thus confounding the effect due to negative feedback. After careful reading of the text, I see that this is not the case, but the terminology is a bit non-intuitive. It would be possible to have quality-dependent positive-only recruitment, and I think that some small changes to the section of the text that*

justifies the introduction of quality dependence (lines 87-90) and/or to table 1 could make things clearer in this regard.

Thank you for your suggestion. We edited the main text of the manuscript, lines 92-95, to specify that the models reported in Table 1 have the parameters set as the best functions obtained through optimisation.

"Table 1 reports the best functions we obtained through numerical optimisation to approximate the target distribution. Including negative feedback inevitably requires a change also in the recruitment function, from constant to linearly proportional to the quality. The difference in the recruitment function between the two models has not been imposed by design but is the result of the numerical optimisation analysis that we show in detail in the Supplementary Text ST1."

Reviewer #2

One suggestion is that the authors consider the recent paper by Bell et al. (2021) showing that response to stop signals changes as a function of colony size and incorporate this in their discussion. It would be interesting to consider this paper in light of Fig. 3 given that the response of colonies of different sizes are, in a way, similar to considering the different stop signalling strength levels shown in this figure. With respect to Fig. 3, I would welcome more discussion or explanation. It seems that the optimal stop signal strength occurs at z=0.4 but that increasing z results in a very large increase in predicted error. Is this a result of using the infinite population model? Would using a finite, more realistic population model yield lower predicted errors as z increases?

Thank you for drawing our attention to Bell et al.'s recent work that is indeed relevant to this study. As suggested by the reviewer, we expanded the discussion of Fig.3 in the main text (lines 135-141) with reference to the recent empirical results by Bell et al.

> Also, the inset of figure 3 with recruitment strength on the x-axis should be mentioned in the legend.

Thank you. As suggested, we included a description of the inset in the caption of Figure 3.

> L155. I suggest, “As a motivating example, decreases in the number of waggle circuit repetitions in honeybee swarms” to clarify that the waggle phase durations are not decreasing, jut the number of waggle dance repetitions.

Thank you, we revised the text as suggested.

Reviewer #3

> The paper is short, but the main findings are apparent in the results section and figures. What is not exactly clear to me is how the model itself brings about the reduction in variance, even in the supplemental information. Perhaps this is primarily a simulation-based result, but it seems like the authors should be able to explain in more detail precisely how the variance-reduction mechanism works. My intuition would tell me that perhaps the additional term is somehow increasing the stability (increasing the amplitude of the main negative eigenvalue) associated with the steady state. Is this something the authors could show for the full stochastic system? Is this something that would appear in a system-size expansion approximation of the full stochastic system? Seems like there should be some analysis that could be done to make it clearer. I would appreciate any response the authors could give to these queries in a revision.

Thank you, this is a good point. We indeed made an analysis of the negative eigenvalues of the two systems, which was included in previous version of the Supplementary Text ST3 (Stability Analysis). We expanded the description

regarding the study of the negative eigenvalues to give more details and further explanation of the different dynamics.

Regarding the stochastic system, we conducted further analysis. We computed the Fokker-Planck equations and from them we derived the first-order and second-order moments of the noise. We studied these equation at convergence ($t \rightarrow \infty$). The equations, which are available in the notebook accompanied with the manuscript, are nonlinear and difficult to solve. Therefore, we applied simplifying approximations to compare the variance of the noise for the two systems. The results that we report in the Supplementary Text ST3 (Stability Analysis) confirm the main result of the paper, predicting a higher variance for the system without negative social feedback.

> Other than this, I would say that the authors could probably move some of the results from the Supplementary Info document up to the main text. It's written rather like a PRL or PNAS article with a lot of interesting analysis tucked into the supplement, but I'd say the sections ST1 and ST3 and their figures could be moved up to the main text.

Thank you for your suggestion, however, we believe that merging the content of the supplementary text with the main text would make the paper less accessible and dilute its impact. The supplementary material contains several equations and is very technical. Our target readership is biologists and previous research has shown that the impact of research papers decreased with the number of equations they contained (Fawcett & Higginson, PNAS 2012). It was suggested that reducing the number of equations would increase the impact of the paper and make it more accessible (Higginson & Fawcett, New J. Phys. 2016). Therefore, if possible, we prefer to maintain our paper concise and more accessible to biologists.

Yours faithfully,

Andreagiovanni Reina
